# Chemically synthesized nanoparticles of iron and iron-carbides

**DOI:** 10.1039/d0ra02996c

**Published:** 2020-08-05

**Authors:** Hafsa Khurshid, Yassir A. Abdu, Eamonn Devlin, Bashar Afif Issa, George C. Hadjipanayis

**Affiliations:** Department of Applied Physics and Astronomy, University of Sharjah Sharjah UAE hkhurshid@sharjah.ac.ae; Research Institute for Medical and Health Sciences (RIMHS), University of Sharjah Sharjah UAE; Dartmouth Hitchcock Medical Center, Department of Radiology Lebanon NH USA; National Center for Scientific Research, Demokritos Greece; Department of Medical Diagnostic Imaging, University of Sharjah Sharjah UAE; Department of Physics and Astronomy, University of Delaware Delaware USA

## Abstract

In this paper, we report a one-pot chemical synthesis technique for the preparation of iron and iron-carbide nanoparticles. Mössbauer spectroscopy, X-ray diffraction and magnetometry were used as the main tools to identify the different phases of Fe–C present. The influence of experimental parameters on the structural and compositional properties of nanoparticles was investigated in detail. These particles show ferromagnetic behavior with room temperature coercivity higher than 300 Oe. The X-ray diffraction was complemented by Mössbauer spectroscopy and thermo-magnetic analysis. Remarkably, the carbon content in iron-carbide nanoparticles (carbon rich or carbon poor iron-carbides) can be modulated simply by varying the experimental conditions, like the reaction time, temperature and iron precursor concentration. Magnetic properties can be tailored based upon crystallographic structure and particles composition.

## Abstract 2

In this paper, we report a one-pot chemical synthesis for the preparation of iron and iron-carbide nanoparticles.

## Graphical abstract

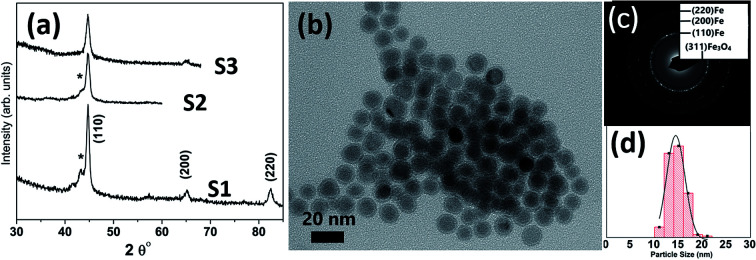

## Introduction

Iron based nanoparticles have been of great interest for researchers for the past few decades because of their wide range applications including data storage, environmental remediation, catalysis, and for diagnosis of diseases and therapy.^1^ Among them, iron oxides and metallic iron have been widely explored.^2,3^ Although, iron oxides are nontoxic in organisms and stable in air/biological environment, their magnetization is low, especially at the nanoscale. Owing to their higher magnetization, metallic iron Fe^(0)^ nanoparticles have been proposed to be better for biomedical applications. However, because of their high surface energy and surface to volume ratio, iron nanoparticles are readily oxidized to form Fe_2_O_3_ when removed from inert atmosphere. Encapsulation of these particles into silica shells has been shown to keep them stable but this caused a significant reduction in saturation magnetization and made them inappropriate for uses where high magnetization is needed. Surface passivation of iron nanoparticles, with or without using oxidizing agents during the chemical reaction, is another approach to make core/shell structures and stabilize these particles.^4^ However, such core/shell nanoparticles are not stable over a long period of time and may become oxidized during ligand exchange to achieve water dispersibility for clinical biomedical applications.^5^

Combining their biocompatibility, high magnetization, and stability for a long period of time, iron-carbide (Fe–C) nanoparticles could make ideal candidates for biomedical applications.^6–8^ Recently, there have been a few reports on the use of cementite (Fe_3_C) as the heating probes for magnetic fluid hyperthermia therapy of cancer tumors.^9^ Recent studies focused on the Fe_3_C@C composites for batteries and electron-catalysis applications. Core–shell Fe_3_C@C nanoparticles have been shown to improve charge transfer, beneficial for inducing active sites for N_2_ adsorption and activation and hence to synthesize high-performance and low-cost electrocatalysts for energy conversion application.^10^ Kou *et al.* claimed that the flexible Fe_3_C/C membrane is a promising candidate for future commercial application of Li–S battery cathode due to its easier large-scale production and lower cost than conventional GO/graphene and carbon nanotube electrode materials.^11^ Physical pyrolysis, sol–gel, sonochemical and laser ablation are among the most common methods for the synthesis of iron carbide nano and micro particles. However, broad size distribution, polydispersity and particle agglomerations are among the biggest challenges for these synthetic techniques. The synthesis of iron carbide nanoparticles by the spray gel technique also introduced broad particle size distribution.^12^ Iron/iron-carbide nanocomposite particles provided higher stability and oxidation resistance when Fe(CO)_5_ was reduced in diphenyl ether at 257 °C.^13^ In a recent report, Meffre *et al.*^14^ have reported the seed mediated fabrication of iron carbide using Fe^(0)^ nanoparticles. Despite a few recent reports, fabrication of monodisperse iron carbide nanoparticles with higher magnetization and narrow size distribution remains a bottleneck for their use in clinical applications.^15^ Moreover, a better control of particle size and composition is essential for the applications of Fe–C particles in catalysis and sensors.

In this work, we report the tailoring of metallic iron nanoparticles into iron-carbide nanoparticles by tuning the reaction conditions during synthesis. Thermal decomposition of Fe(CO)_5_ is a well known method to synthesize monodisperse iron Fe^(0)^ nanoparticles with well controlled size and shape. Albeit, Fe(CO)_5_ thermal decomposition is amongst common method to fabricate iron-carbide nanoparticles. In this paper, we investigate reaction conditions in detail to describe the fabrication of Fe^(0)^*versus* Fe–C during chemical synthesis. We show that it is possible to obtain Fe^(0)^, Fe_2_C, Fe_3_C and meta-stable Fe_*x*_C by simply varying the reaction conditions during Fe(CO)_5_ thermal decomposition. The reaction time, temperature and precursor concentration are found to be key factors to modulate nanoparticles composition and crystallographic structure and hence magnetic properties.

## Experimental procedure

The particle synthesis was carried out using commercially available reagents without further purification. Iron pentacarbonyl (Fe(CO)_5_), oleylamine (OY, 70%), 1-octadecene (90%), oleic acid (OA, 99%) were purchased from Sigma Aldrich.

In a typical synthesis, 0.3 mM of OY and 0.32 mM of OA were dissolved in 60 mM octadecene in a three neck flask and heated at elevated temperatures (up to 120 °C) in an air-free atmosphere while continuously purging with Ar + 5% H_2_ to remove any free oxygen dissolved in the solvent and surfactants. Subsequently, the temperature was raised to 270 °C and Fe(CO)_5_ was injected steadily under vigorous stirring. A white smoke accompanied by the black colored reaction mixture, immediately after injection, indicates a successful decomposition of Fe(CO)_5_ and the particles formation. The reaction temperature rises to a few degrees (∼2 to 3 °C) because of the exothermic nature of the reaction. The reaction mixture was allowed to cool down to room temperature by removing the heating mantel. The dark nanoparticles solution was precipitated by addition of absolute ethanol, separated by a strong laboratory magnet and then dispersed in hexane.

X-ray diffraction (XRD), using a Rigaku Ultima IV X-ray diffractometer with CuKα radiation, was used for identification of the crystalline phases in the nanoparticle samples. JEM 3010 TEM by JEOL was used to characterize the microstructure and size of the nanoparticles. Magnetic measurements were made using QD Versalab 3-Tesla vibrating sample magnetometer. Mössbauer spectroscopy measurements at 80 K were done in transmission geometry using a conventional constant-acceleration spectrometer, with the source [^57^Co(Rh)] kept at room temperature (RT). Sample temperature was controlled using a Janis cryostat, and spectra were analyzed using a least-squares method with Lorentzian lineshapes. The central shift (CS) is given relative to α-Fe at RT.

## Results and discussion

**Fig. 1:**
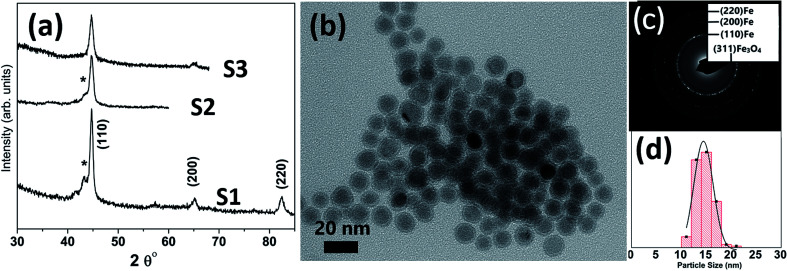
(a) XRD micrographs of nanoparticles synthesized at different concentrations of iron precursor (S1) 3.7 μm, (S2) 1.5 μm, and (S3) 0.22 μm; the shoulder (*) is associated with Fe–C (b) TEM image of sample ‘S3’ with (c) showing its selected area diffraction pattern (SAD) and (d) particle size distribution 14.4 ± 1.1 nm.

**Fig. 2:**
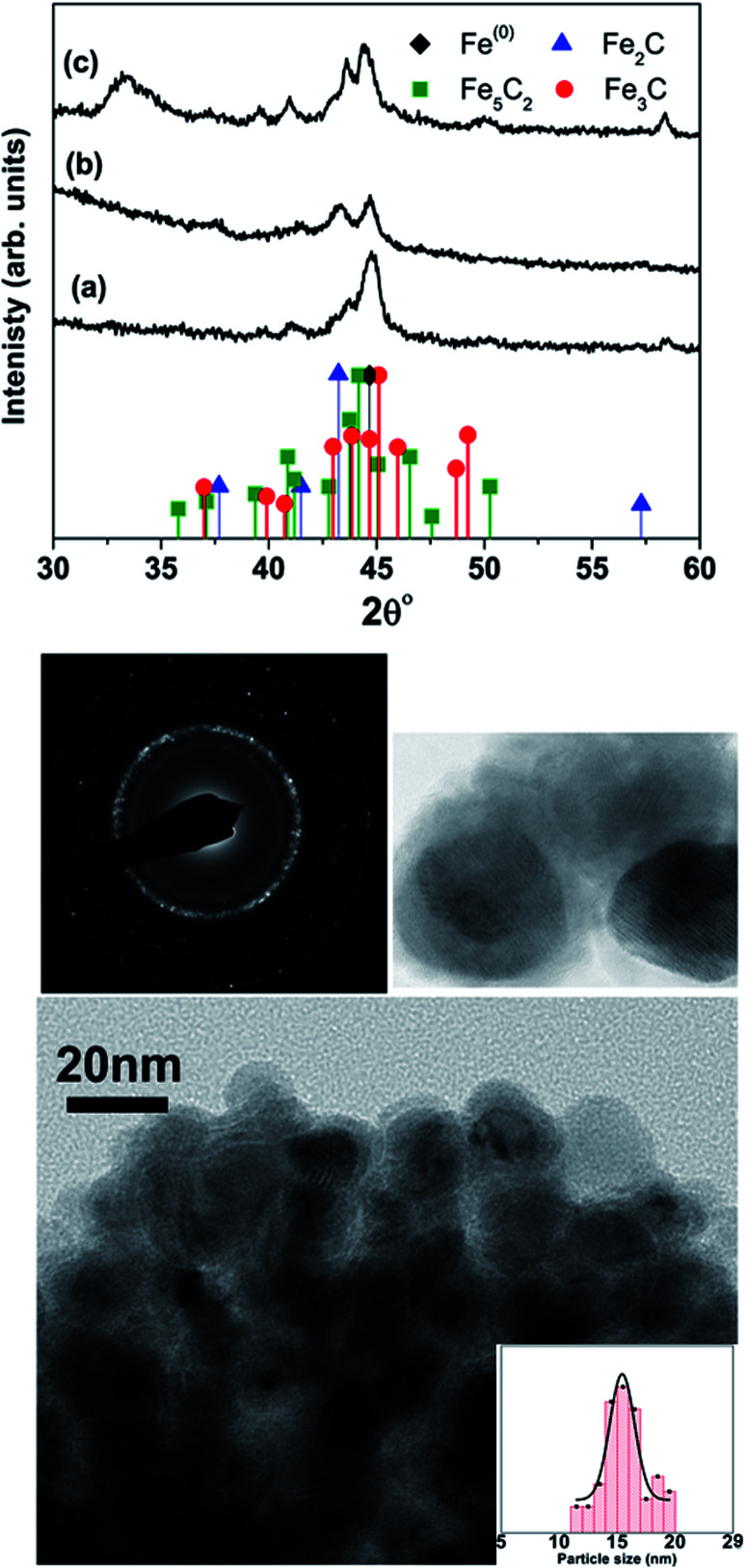
XRD micrographs of samples prepared at 300 °C with a refluxing time (a) 10 min, (b) 1 h, and (c) 3 hours. The TEM image is from sample ‘S6’ with the inset showing high-resolution image and SAD and particle size distribution of 15.5 ± 1.2 nm.

Table 1 lists the samples and reaction conditions used in this study. TEM images of nanoparticles prepared at 275 °C with minimal refluxing time and lowest Fe(CO)_5_ concentration, are presented in Fig. 1. These particles are spherical with a darker core and lighter shell, revealing their core/shell like morphology with an average size 15.2 ± 1.1 nm (estimated by counting above 300 particles in TEM images). Such a core/shell morphology has been reported earlier in these reactions, where the core is composed of Fe^(0)^ and the shell is an iron oxide; either magnetite or maghemite.^16,17^ HRTEM clearly showed uniform lattice fringes of the core, corresponding to (110) bcc iron with the shell composed of randomly oriented grains of iron oxide. The Fig. 1c shows selected area diffraction from the image where all the diffraction rings match the reflections from bcc Fe^(0)^. To assess the crystallographic phases present in these particles, X-ray diffraction patterns were collected from samples prepared with different concentrations of Fe(CO)_5_ while keeping the reaction time minimum for all samples. The reaction mixture was cooled down to room temperature immediately after the injection of Fe(CO)_5_ to minimize the refluxing/reaction time. For sample S1, where the iron precursor (Fe(CO)_5_) concentration is 3.7 μM, the diffraction peaks at 44.7, 65 and 82° correspond to the characteristic reflections (110), (200) and (220), respectively, of bcc Fe^(0)^ (Fig. 1a). The obvious peaks corresponding to (110) and (200) reflections of bcc Fe^(0)^ in the 0.22 μM concentration of Fe(CO)_5_, sample S3, agree well with the SAD of this sample (Fig. 1). In addition to the bcc Fe^(0)^ peaks, there is a shoulder peak at 43.5°, whose intensity is decreasing with decreasing iron precursor concentration (Fig. 1a). This shoulder peak may indicate the presence of Fe–C phases in the samples. It is well known that in thermal decomposition reactions of organometallic compounds, the particle size and composition of the particles depend on the reaction temperature and time, and surfactant concentrations.^18,19^ Particle size is related to iron nanocluster formation during nucleation step that in turn depends upon precursor-to-surfactant ratio and heating rate affecting nucleation and concentration of nuclei after the burst nucleation phase.^18^ The reaction temperature and surfactant concentration were kept same for these samples. A careful analysis from TEM imaging manifested that particle size does not change much with reaction time (size distribution mentioned in Table 1), that in turn indicates that nucleation and growth steps are very robust and rapid. However, new peaks appearing in XRD micrograph may indicate crystallographic and compositional changes in particles with reaction time. To investigate the origin of this emerging peak, a temperature of 300 °C was chosen to study the effect of reaction time on the nanoparticles, keeping the Fe(CO)_5_ concentration the same (3.7 μM). Fig. 2a shows the XRD patterns of samples with different refluxing periods of 10 min, 60 min and 180 min (samples S4, S5, and S6, respectively). Along with the (110) characteristic reflection from bcc iron (PDF no. 75-0444, JCPDS card no. 2004) at 44.7°, the peaks at ∼39.5°, 41° and 43.6° became more pronounced with increased refluxing time. These peaks match the characteristic reflections of non-stoichiometric iron carbide phases like Fe_3_C and Fe_5_C_2_ (JCPDS card no. Fe_3_C 01-089-2005, and Fe_5_C_2_ phase JCPDS card no. 51-0997, respectively). It is concluded that higher reflexing temperature and longer refluxing time caused the formation of non-stoichiometric iron-carbide phases in these samples. Interestingly, refluxing time did not affect the average particle size. However, HRTEM images revealed a set of darker and lighter fringes in these particles. Such fringes are typical in particles with crystallographic strain that is common in iron-carbides. Rawers *et al.*^20^ proposed the hypothesis for the formation of bct Fe–C in an Attritor ball milled iron powder along with 2% carbon. At first, bct iron is formed when mechanically infused carbon went into interstitial octahedral sites distorting one of the faces of the bcc lattice into a local bct structure, greatly increasing the local lattice strain. As the reaction continued, carbon atoms form local clusters producing a highly distorted and strained lattice. The rapid diffusion of carbon into local clusters resulted in an ordered structure of Fe and C. However, a solid solution of iron and carbon is formed in the mechanically processed (without high-energy ball milling) iron powder along with carbon. The presence of carbon (C) in the particles can be attributed to the subsequent diffusion of C from CO and surfactants into the iron clusters at high temperatures. Iron carbide nanoparticle synthesis has been reported before by chemical vapor condensation after decomposing Fe(CO)_5_ in the presence of CH_4_ at high pressure and temperature.^13^ Lee *et al.*^17^ have reported Fe–C particle synthesis above 101 kPa, as the residence time of vapor molecules depends upon the pressure inside. At lower pressure, the residence time of vapors to react with CH_4_ to form Fe–C is not enough to react and form Fe–C phases. In our synthesis, we believe that during high temperature injection of iron precursor in a closed vessel, the pressure increases inside the reaction flask because of high volatility of Fe(CO)_5_ at high temperatures. It is believed that in case of low concentration of Fe(CO)_5_, the pressure inside the flask is not high enough for C, from CO vapors, to diffuse into Fe. Moreover, a limited availability of CO vapors at lower Fe(CO)_5_ concentration also suppressed the Fe–C formation and the sample composition is dominated by Fe^(0)^.

**Table 1:** Samples and reaction conditions used in this study

Sample	Iron precursor conc. (μM)	Reaction time (min)	Reaction temperature (°C)	Magnetization (emu g^−1^)	Particle size distribution (nm)
S1	3.7	0	275	124	13.3 ± 1.6
S2	1.5	0	275	130	15.4 ± 1.2
S3	0.22	0	275	135	14.4 ± 1.9
S4	3.7	10	275	126	13.8 ± 1.2
S5	3.7	60	275	110	13.9 ± 2.7
S6	3.7	180	300	90	15.5 ± 1.2

**Fig. 3:**
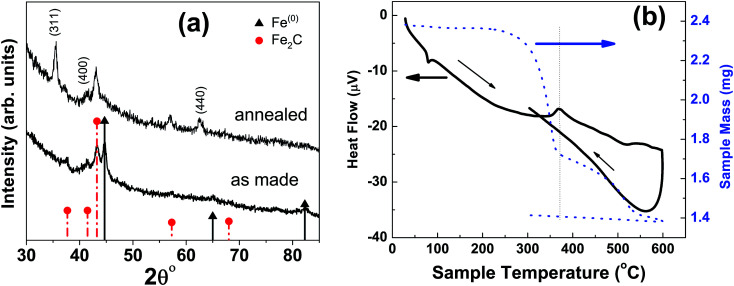
(a) XRD micrograph of as-made and annealed nanoparticles at 300 °C with a refluxing time of one hour; sample ‘S5’ in Fig. 2. (b) The new peaks in annealed sample correspond to the magnetite characteristic reflections. The image on the right is thermo-gravimetric analysis (TGA) and differential thermal analysis (DTA) of particles under free flow of argon.

**Fig. 4:**
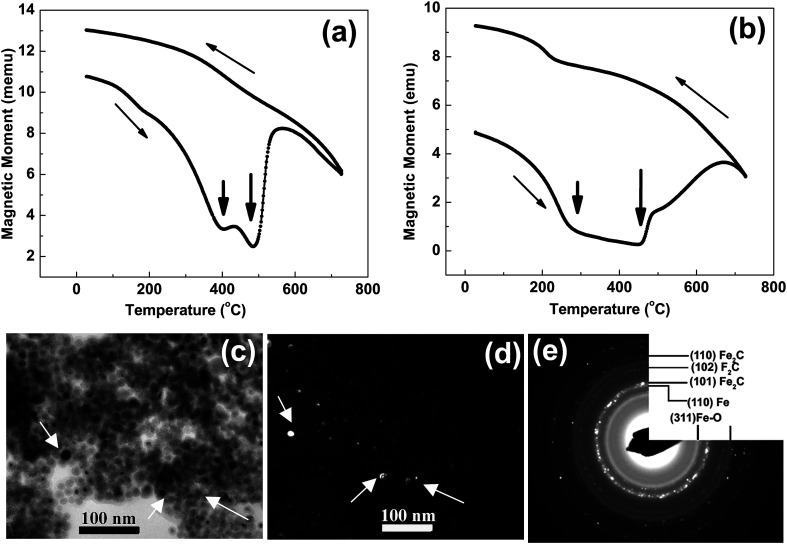
(a and b) show the magnetization *versus* temperature measurements on samples ‘S5’ and ‘S6’ in Fig. 2. The micrograph (c) is a bright field TEM image and (d) dark field TEM image from the most intense diffraction ring (the second ring) in SAD (e) of sample ‘S5’ in Fig. 2.

At higher concentration of iron precursor (sample S5), the Fe_2_C hexagonal phase appeared, as indicated by XRD. Fig. 3a shows the XRD patterns of sample S5 nanoparticles before and after annealing at 500 °C for one hour. In the as-made sample the peak at 44.7° corresponds to the (110) reflection of bcc iron with a grain size of 9.9 nm (calculated using Scherer's equation) whereas the intense peak at 43.2° with 8.4 nm grain size may correspond to the (101) reflection of Fe_2_C. To identify the magnetic phases in this sample, particles were heated from 40 to 600 °C at a rate of 10 °C min^−1^ under free flow of argon in a TG-DTA. The existence of exothermic peaks at ∼370 °C (Fig. 3b) indicates the presence of meta-stable phases. This temperature corresponds to the Curie temperature of hexagonal Fe_2_C phase,^21^ consistent with the XRD results of this sample. Upon annealing under vacuum at 500 °C for one hour, Fe_2_C still remained there with the same grain size but the amount of oxides increased in the sample at the expense of iron (Fig. 3a). These results indicate the existence of separate Fe and Fe–C nanoparticles in the sample. To compliment the XRD and thermo-gravimetric results, thermomagnetic measurements (Fig. 4a) performed on this sample (at a rate of 5 °C min^−1^ under 10 kOe applied field), clearly indicate a Curie temperature at around 370 °C which corresponds to the Fe_2_C hexagonal phase.^21^ However, the rapid increase in magnetic moment after 400 °C might be an indication of the appearance of another phase or a structural transformation. It has been reported before that γ-Fe_2_O_3_ transforms irreversibly to α-Fe above 400 °C^13^ and this may increase the magnetic moment. Furthermore, Fe–C phases are metastable phases and they can decompose into α-Fe and C residues at high temperature. To locate the Fe_2_C specifically among nanoparticles, we have performed dark field TEM imaging in these samples. The diffraction ring corresponding to (101) planes of Fe_2_C in the SAD pattern was used to obtain the dark field image (Fig. 4). As a direct result, it is possible to see the Fe_2_C phase in the nanoparticles very clearly. The brighter regions in the dark-field image, marked by arrows are the Fe_2_C particles, among the darker Fe/Fe–O core shell particles. On the other hand, the thermomagnetic measurements of samples prepared at longer refluxing time of 180 min (sample 6), Fig. 4b, showed a Curie temperature of 240 °C due to the presence of cementite Fe_3_C.^22^ A rapid increase in magnetic moment after 400 °C might indicate the decomposition of the metastable Fe–C phases to α-Fe and C at high temperature. Hägg carbide (Fe_2_C) is also known as the intermediate stage of the formation of Fe_3_C. During the Fischer Tropsch synthesis process, if reaction is continued further at high temperature, or more Fe^(0)^ is provided, Fe_3_C will be the final reaction product.

**Fig. 5:**
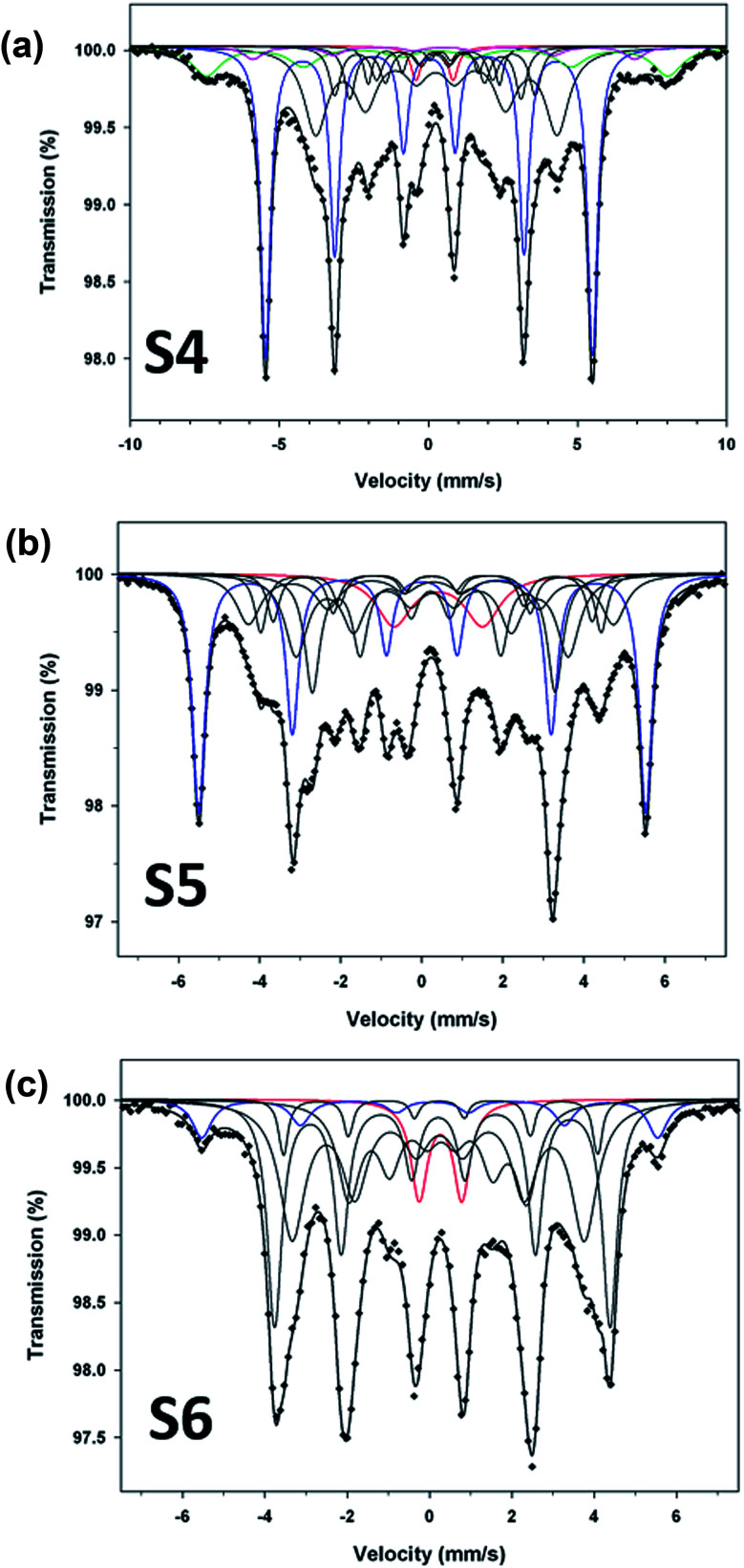
Mössbauer spectra (at 80 K) for samples S4 (a), S5 (b) and S6 (c). Blue sextet: α-Fe; black sextets: Fe–C; green sextet: magnetite; pink sextet: goethite; red doublet: Fe^3+^ phase.

To compliment XRD results and determine the relative proportions of Fe-containing phases, Mössbauer spectroscopy was performed on samples S4, S5 and S6. The spectra collected at 80 K are shown in Fig. 5, and the Mössbauer parameters are given in Table 2. They are fitted, using Lorentzian line shapes, to a combination of a quadrupole doublet and magnetic sextets. The quadrupole doublet, with a relative area ranging from 2% to 9% (Table 2), may be assigned to an Fe^3+^ phase; possibly a superparamagnetic Fe oxide/hydroxide. The sextet with a hyperfine magnetic field of ∼34 T and near zero QS is characteristic of α-Fe. The relative area of this sextet decreases from 40% (for sample S4) to 7% (for sample S6), *i.e.* with increase of the reaction time, in accordance with the XRD results. The sextets with smaller values of hyperfine magnetic fields (Table 2) are attributed to the Fe–C phases.^23^ The spectrum of sample S4 (Fig. 4a), which has the largest proportion of α-Fe (40%), contains two additional sextets with *H* = 48.0 T and 39.7 T that could be assigned to Fe_3_O_4_/γ-Fe_2_O_3_ (11%) and α-FeOOH (3%), respectively (Table 2). X-ray diffraction analysis of sample S5 (Fig. 5b) indicated the presence of α-Fe and hexagonal Fe_2_C (ε-Fe_2_C). The latter phase is isostructural with ε′-Fe_2.2_C and along with Fe_*x*_C they form the O carbides, where C atoms occupy octahedral interstices in hcp an Fe lattice. Following the Mössbauer work of Le Caer *et al.*^23^ and Liu *et al.*^24^ on O carbides, we assign the two Fe–C sextets with *H* = 26.0 and 18.6 T in the spectrum of sample S5 to ε-Fe_2_C, and those with *H* = 27.9, 24.4 and 20.8 T to a mixture of ε′-Fe_2.2_C and Fe_*x*_C phases (Table 2). For sample S4, the *H* values of the Fe–C sextets (Table 2) indicate the presence of a mixture of O carbides and χ-Fe_2_C_5_ (*H* = 13.7 and 25.1 T).^21^ The spectrum of sample S6 (Fig. 5c) is dominated by Fe–C phases, accounting for 86% of total Fe, and the Fe–C sextets in this sample are due to a mixture of χ-Fe_2_C_5_ (*H* = 13.4, 22.0 and 25.3 T) and θ-Fe_3_C (*H* = 23.7 T) phases.^25^

**Table 2:** Mössbauer parameters (at 80 K) of samples S4, S5 and S6^a^

Sample	Fe site/phase	CS (mm s^−1^)	QS (mm s^−1^)	*H* (T)	*A* (%)
S4	Fe^3+^-phase	0.21	1.25	—	2
	α-Fe	0.03	−0.01	34.0	40
	χ-Fe_2_C_5_	0.26	0.02	25.1	26
		0.29	−0.10	13.7	4
	ε′-Fe_2.2_C	0.22	0	20.8	7
	ε-Fe_2_C	0.23	0.01	17.8	7
	Fe-oxide	0.29	0*	48.0	11
	Fe-oxide	0.52	0*	39.7	3
S5	Fe^3+^-phase	0.41	2.19	—	9
	α-Fe	0.01	0.01	34.2	30
	ε′-Fe_2.2_C	0.26	−0.03	27.9	12
		0.25	0.02	24.4	6
		0.26	0	20.8	18
	ε-Fe_2_C	0.24	−0.01	26.0	7
		0.25	0.04	18.6	18
S6	Fe^3+^-phase	0.27	1.03	—	7
	α-Fe	0.04	−0.03	34.3	7
	χ-Fe_2_C_5_	0.26	0.05	25.3	30
		0.23	−0.01	22.0	31
		0.24	−0.04	13.4	20
	θ-Fe_3_C	0.25	0.01	23.7	5

a CS = centre shift (±0.02 mm s^−1^), QS = quadrupole splitting (±0.02 mm s^−1^), *H* = hyperfine magnetic field (±0.5 T), *A* = relative area (±3%). * fixed parameter. See text for details.

The Mössbauer results indicate that reaction time plays an important role in the formation of Fe–C phases. The Mössbauer spectra (Fig. 5) clearly show the increase of subspectra due to Fe–C phases with increase of the reaction time. The percentages of Fe–C phases in samples S4, S5 and S6 are 44%, 61% and 86%, respectively (Table 2), which implies that the carbon content in these samples increases in that direction.

**Fig. 6:**
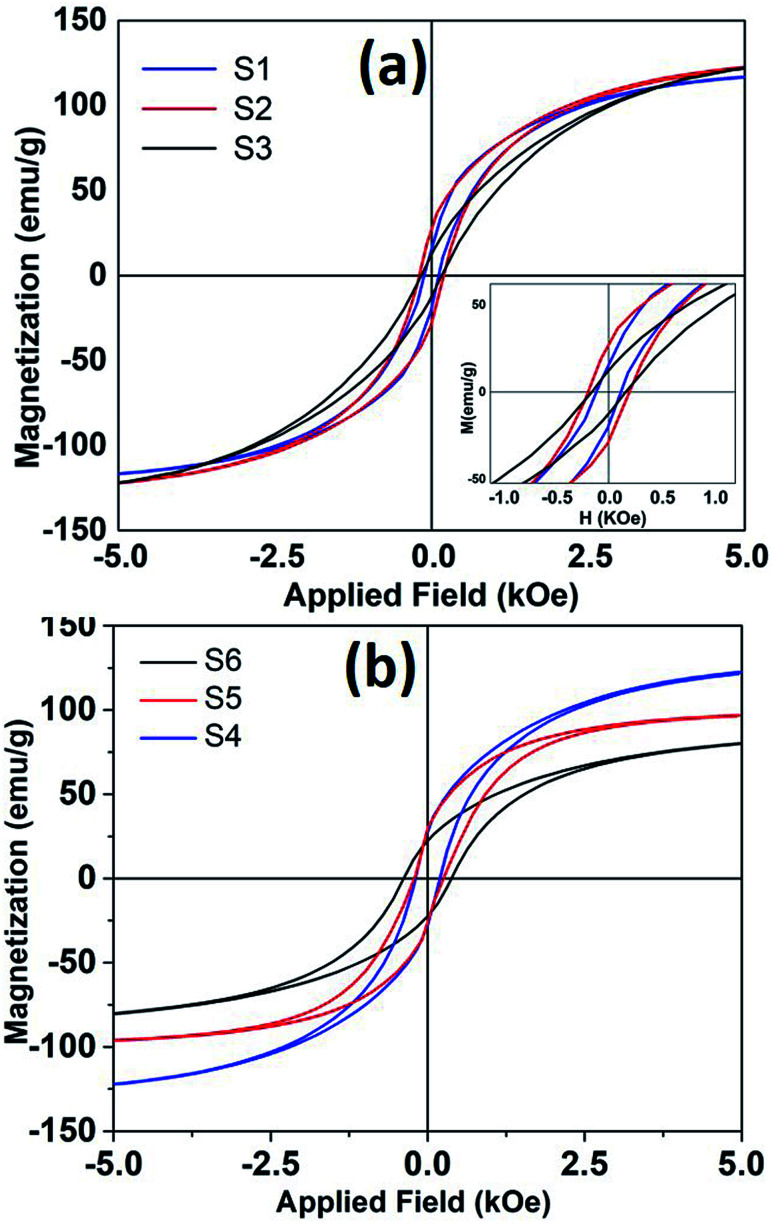
The room temperature hysteresis loops of nanoparticles synthesized at different refluxing temperatures. (a) Sample S1, S2 and S3 and (b) shows hysteresis loops for samples S4, S5 and S6.

Any changes in the particles composition and crystallographic structure should directly influence their magnetic properties. DC hysteresis loops, for all the samples is shown in Fig. 6. The room temperature saturation magnetization (estimated from law of approach to saturation^26^) and coercivity found to be varied for different samples depending upon reaction conditions.

It is seen that magnetization decreases and coercivity increase when reaction was carried out for longer time. For sample S4, when reaction time was only 10 minutes, coercivity is 200 Oe that increased to 382 Oe with a reaction time of 3 hours. Coercivity is below 120 Oe when reaction time and Fe(CO)_5_ concentration kept minimum (sample S1). It is to be reminded that higher iron precursor concentration and longer reaction times helps to incorporate more carbon content in nanoparticle, as seen from XRD, and Mössbauer data. The presence of high anisotropy iron-carbide phases could explain the enhanced coercivity in these samples. Moreover, room temperature magnetization also decreases with higher carbon content. The room temperature *M*_s_ for bulk iron is about 217 emu g^−1^. The maximum saturation magnetization estimated in our samples is 135 emu g^−1^. The presence of metastable Fe_*x*_C phases can also dilute the magnetization of Fe nanoparticle samples. As mentioned earlier, the particles with maximum Fe^(0)^ content showed a core/shell like morphology, where core if iron and shell is composed of small grains of iron-oxide. Such a structure/morphology is well known to incorporate higher surface and intra-grain spin disorder^27^ that in turn decreases particle magnetization.

## Conclusions

Nanoparticles of iron and iron-carbides have been synthesized chemically by thermal decomposition of Fe(CO)_5_ at high temperature. Refluxing for a long time does not affect much the average particle size: however, it changes their composition and crystallographic structure. The Fe–C phases were identified by measuring the Curie temperature of samples and were further complimented by XRD and Mössbauer spectroscopy. The XRD analysis and Mössbauer spectroscopy of these samples indicate that there is an obvious increase in the iron carbide phases with increase of Fe(CO)_5_ concentration and reaction time. These nanoparticles show ferromagnetic behavior with room temperature coercivity higher than 300 Oe. The magnetic properties can be modulated depending upon sample crystallographic structure and composition. In summary, the reaction conditions were investigated in detail to describe the fabrication of Fe^(0)^*versus* Fe–C during chemical synthesis. The reaction time and precursor concentration are the key factors to modulate/control nanoparticles composition and crystallographic structure. Future work will be focused on obtaining single-phase Fe–C nanoparticles with high iron content (Fe_3_C) and study their cytotoxicity behavior.

## Conflicts of interest

There are no conflicts to declare.

## Supplementary Material
